# Veterinary Education in England

**Published:** 1881-01

**Authors:** 


VETERINARY EDUCATION IN ENGLAND cannot be in such a vastly superior condition to that in this city, if we may judge by the following quotation from an editorial in the *Veterinary*

*Review.* The editor says: "We cannot be accused of exaggeration when we venture to assert that scores of young men, very imperfectly educated—scarcely able to read and write their own language—who may have failed in other pursuits, and are without any idea of the requirements of a veterinary surgeon, have entered our schools, and in due time received their diploma without ever having given a dose of medicine, performed the slightest operation, or gained the slightest insight into the ordinary routine practice."

There is a very active discussion going on, in the same journal, as to whether the English veterinary students keep themselves as clean as they ought. This important matter has not yet been decided.

Such comment and discussion as the above cannot occur in the colleges of this city, at least. We should add, perhaps, that the Royal College of Veterinary Surgeons has recently resolved that no student shall receive a diploma without having had a year's pupilage.

